# Parallel Evolution under Chemotherapy Pressure in 29 Breast Cancer Cell Lines Results in Dissimilar Mechanisms of Resistance

**DOI:** 10.1371/journal.pone.0030804

**Published:** 2012-02-02

**Authors:** Bálint Tegze, Zoltán Szállási, Irén Haltrich, Zsófia Pénzváltó, Zsuzsa Tóth, István Likó, Balázs Győrffy

**Affiliations:** 1 1st Department of Pediatrics, Semmelweis University, Budapest, Hungary; 2 Children's Hospital, Boston, Massachusetts, United States of America; 3 Center for Biological Sequence Analysis, Technical University of Denmark, Kongens Lyngby, Denmark; 4 2nd Department of Pediatrics, Semmelweis University, Budapest, Hungary; 5 Gedeon Richter Plc., Budapest, Hungary; 6 Laboratory of Functional Genomics, Charité Universitätsmedizin, Berlin, Germany; 7 Research Laboratory of Pediatrics and Nephrology, Hungarian Academy of Sciences, Budapest, Hungary; University Health Network, Canada

## Abstract

**Background:**

Developing chemotherapy resistant cell lines can help to identify markers of resistance. Instead of using a panel of highly heterogeneous cell lines, we assumed that truly robust and convergent pattern of resistance can be identified in multiple parallel engineered derivatives of only a few parental cell lines.

**Methods:**

Parallel cell populations were initiated for two breast cancer cell lines (MDA-MB-231 and MCF-7) and these were treated independently for 18 months with doxorubicin or paclitaxel. IC50 values against 4 chemotherapy agents were determined to measure cross-resistance. Chromosomal instability and karyotypic changes were determined by cytogenetics. TaqMan RT-PCR measurements were performed for resistance-candidate genes. Pgp activity was measured by FACS.

**Results:**

All together 16 doxorubicin- and 13 paclitaxel-treated cell lines were developed showing 2–46 fold and 3–28 fold increase in resistance, respectively. The RT-PCR and FACS analyses confirmed changes in tubulin isofom composition, TOP2A and MVP expression and activity of transport pumps (ABCB1, ABCG2). Cytogenetics showed less chromosomes but more structural aberrations in the resistant cells.

**Conclusion:**

We surpassed previous studies by parallel developing a massive number of cell lines to investigate chemoresistance. While the heterogeneity caused evolution of multiple resistant clones with different resistance characteristics, the activation of only a few mechanisms were sufficient in one cell line to achieve resistance.

## Introduction

Breast cancer chemotherapy resistance is a complex multifactorial problem, where several pathways may act simultaneously and influence each other leading to failure of systemic treatment [1]. Doxorubicin and paclitaxel are used in recurrent or metastatic breast cancer as a single agent or in combination (http://www.nccn.org).

A principal mechanism of action of anthracyclines is their ability to intercalate into DNA. DNA-bound anthracycline binds DNA topoisomerase II, inducing DNA cleavage in an ATP-dependent manner [2]. Anthracycline resistance might be mediated through overexpression of P-glycoprotein [3], [4], [5], lung resistance protein [6] and multidrug-resistance proteins [7], [8], proteasome subunits [9], increases in antioxidant defenses [10], alterations in apoptotic signaling and TOP2 activity [5], [11], [12]. Most recently, over-expression of genes in the chromosomal region 8q22 have been shown to be associated with anthracycline resistance. One of those genes, LAPTM4B, sequesters anthracyclines away from the nucleus, the location of their therapeutic action [13]. Taxanes act on the microtubuli and resistance against them might be mediated through expression changes and mutations in ABC transporters like MDR1 [1], [14], [15], beta-tubulin isoforms [16], [17], tubulin mutations [18] and mictotubule-associated protein tau [19]. Identification of resistance mechanisms holds the potential of developing biomarkers that can predict disease outcome following treatment with specific chemotherapeutic agents.

There are several alternative strategies to develop chemotherapy response predictors, none of them being ideal, each having its own advantages and disadvantages. Genome scale molecular profiling, such as microarray analysis of tumor biopsies has the potential of most faithfully characterizing the molecular changes associated with response to therapy in the primary tumors [20], [21]. However, due to the large number of variables (tens of thousands of measured genes) relative to the limited number of patients in those cohorts (a few hundred at most) such studies are prone to overfitting [22]. A possible way to circumvent this issue is identifying the potentially relevant biomarkers or mechanisms from either prior biological knowledge coupled with bioinformatics analysis [23], or from appropriate model systems such as cancer cell lines. A breast cancer specific follow up on the initially optimistic studies using cancer cell lines [24], [25], however, failed to identify clinically validated biomarkers. For example, a clinical evaluation of chemotherapy response predictors developed from breast cancer cell lines was performed recently [26]. In this study, nineteen cell lines were used to derive predictors of response to paclitaxel (T), 5-fluorouracil (F), doxorubicin (A) and cyclophosphamide (C). Then, the signatures were used to classify over hundred patients who received preoperative TFAC chemotherapy. However, there was no significant correlation between response and predicted response for either individual or for combined predictors.

An apparent problem with this and earlier studies was the use of a panel of highly heterogeneous cell lines for the identification of the predictors. Developing resistant cell lines to a given agent and then comparing the resistant and the sensitive parent cell line may circumvent this problem. For example MET inhibitors were recently investigated by Qi et al [27] by the comparison of a gastric carcinoma cell line and three resistant sub–cell lines. They found at least two mechanisms of resistance that arose simultaneously and they also observed the capability of a single cancer cell to develop multiple, distinct resistance mechanisms simultaneously. Similar results were observed in lung cancer patients resistant to EGFR inhibitors [28], [29].

In the present study we went one step further in the simulation of the evolution of chemotherapy resistance development in a cell culture by significantly increasing the number of parallel developed cell lines. We assumed that truly robust and relevant resistance mechanisms will emerge in multiple cell lines and a clinically relevant convergent pattern of resistance can be identified. Twenty-nine subpopulations of two breast cancer cell lines were separated and treated with increasing concentrations of doxorubicin and paclitaxel for 18 months. These cells were then investigated to explore whether known resistance mechanisms consistently emerge and therefore the same strategy could be used to identify novel mechanisms as well.

## Materials and Methods

### Cell culture maintenance

The human breast cancer cell lines MCF-7 and MDA-MB-231 were obtained from ATCC, they were cultured in Leibovitz L-15 medium (Invitrogen) supplemented with 10% fetal calf serum (Invitrogen), 2.5 mg/l transferrin, 1.1 g/l NaHCO_3_, 1% minimal essential vitamins, and 20 000 kIE/l trasylol in a humidified atmosphere containing 5% CO_2_ at 37°C.

### Development of resistant cell populations

Ten sub-populations of each cell line for each of the two drugs were separated. The new cell lines were generated parallel by treatment with gradually increasing concentration of doxorubicin (EBEWE Pharma, Unterach, Austria) or paclitaxel (Sigma-Aldrich, Steinheim, Germany). After each week, the confluence was assessed: for cells lines with confluence below 50%, the treatment was stopped, for confluence between 50–70%, the treatment was maintained and for those with a confluence over 70% a portion of the cells was frozen. After 3 weeks of growth at a specific dose of treatment, the concentration was increased. The increase was in 10 fold increments until we reached 0.1× the clinical dose, then 0.3× and 0.6× followed. Before drug concentration increase an MTT cell proliferation assay (Cell Proliferation Kit I (MTT) Roche, IN, USA) was performed to monitor sensitivity of the resistant cell lines. Additionally, vehicle-treated parental cell lines were kept in culture for the duration of the study and these were used as control cell lines.

### Measurement of cross-resistance

Cross-resistance measurements were performed on the parental and the resistant cell lines. IC_50_ values were determined for cisplatin (Sigma-Aldrich, Steinheim, Germany), doxorubicin, paclitaxel and 5-fluorouracil (Sigma-Aldrich, Steinheim, Germany) using the MTT Cell Proliferation Kit (Roche, Mannheim, Germany) and GraphPad Prism software.

### TaqMan RT-PCR measurements

TaqMan real-time PCR was used to measure the expression of selected genes using an Applied Biosystems Micro Fluidic Card System in 31 samples (27 resistant sub-lines and both parental cell lines in duplicates). The measurements were performed using an ABI PRISM® 7900HT Sequence Detection System as described in the products User Guide (http://www.appliedbiosystems.com, CA, USA). The genes were selected based on a literature search, to include genes correlated to doxorubicin and paclitaxel resistance, breast cancer hormone receptors and survival associated genes. The complete list of genes and TaqMan IDs are listed in **Table S1**. For data analysis the SDS 2.2 software was used. The extracted delta Ct values were normalized to the average of 3 different housepeeking genes (18S, HPRT1 and RPLP0). Spearman rank correlation was computed to compare the expression for the genes and the resistance against doxorubicin and paclitaxel. Step-up multiple testing correction was performed [30] and statistical significance was set at p = 0.05. For the visualization of the results, hierarchical clustering was performed using the Genesis software.

### Flow cytometric analysis

The ability of the cells to carry out of Pgp-mediated efflux was assessed by using the Pgp substrate rhodamine 123. Cells were seeded in 6-well plates at a density of 5×10^5^ cells and allowed to attach overnight. Doxorubicin or paclitaxel (for the resistant cell lines, respectively) were added to the cells and incubated for 0.5 h, then rhodamine 123 (10 µM) was added to the cells and after incubation the cells were collected and washed with fresh medium and resuspended in normal growth medium without doxorubicin/paclitaxel or rhodamine 123. Then the cells were centrifuged and washed in ice-cold PBS. At last, the cells were resuspended in 1 ml PBS and analyzed by flow cytometry. Fluorescence was measured from 10^4^ cells and cell-count was plotted against rhodamine intensity.

### Conventional cytogenetics

After the end of the development, treated and vehicle-treated cell lines were cultured for 3–5 days. When the monolayer cultures showed ∼80% confluence, they were incubated overnight with 0,02–0,06 µg/ml final concentration of Colcemid. After detaching the cells with 1× trypsin/EDTA, chromosomal preparations were made according to standard techniques that used 0,067 M KCl as hypotonic treatment and fixation in methanol/acetic acid. Chromosome analysis was performed on metaphase cells G-banded with trypsin and Wright Giemsa stain. Ten metaphases were evaluated for each sample with an Axioskop 2 Mot Plus microscope (Carl Zeiss GmbH, Germany) and Cytovision 3.6 or Mac Ktype 5.6 computer analysis system for karyotyping (Scientific Systems, UK). Karyotypes were described according to the International System for Human Cytogenetic Nomenclature (ISCN 2009).

### Chromosomal instability

Chromosomal instability (CIN) was measured by counting the average number of variations of chromosomes 3, 17 and 21 in ten different metaphases. Then, the Shannon Diversity Index (H) was computed using the formula [31]:
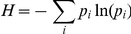
where *pi* is the frequency of a given number of a chromosome *(i)* in the cell lines. Thus, H was used to estimate the degree of numerical chromosomal heterogeneity within each of the developed resistant cell lines. Cell lines having an index at least 2.1 were designated as CIN positive (CIN+) tumors and remaining cell lines were designated as CIN negative (CIN−) tumors.

## Results

### Development of resistant cell populations

**Figure 1:**
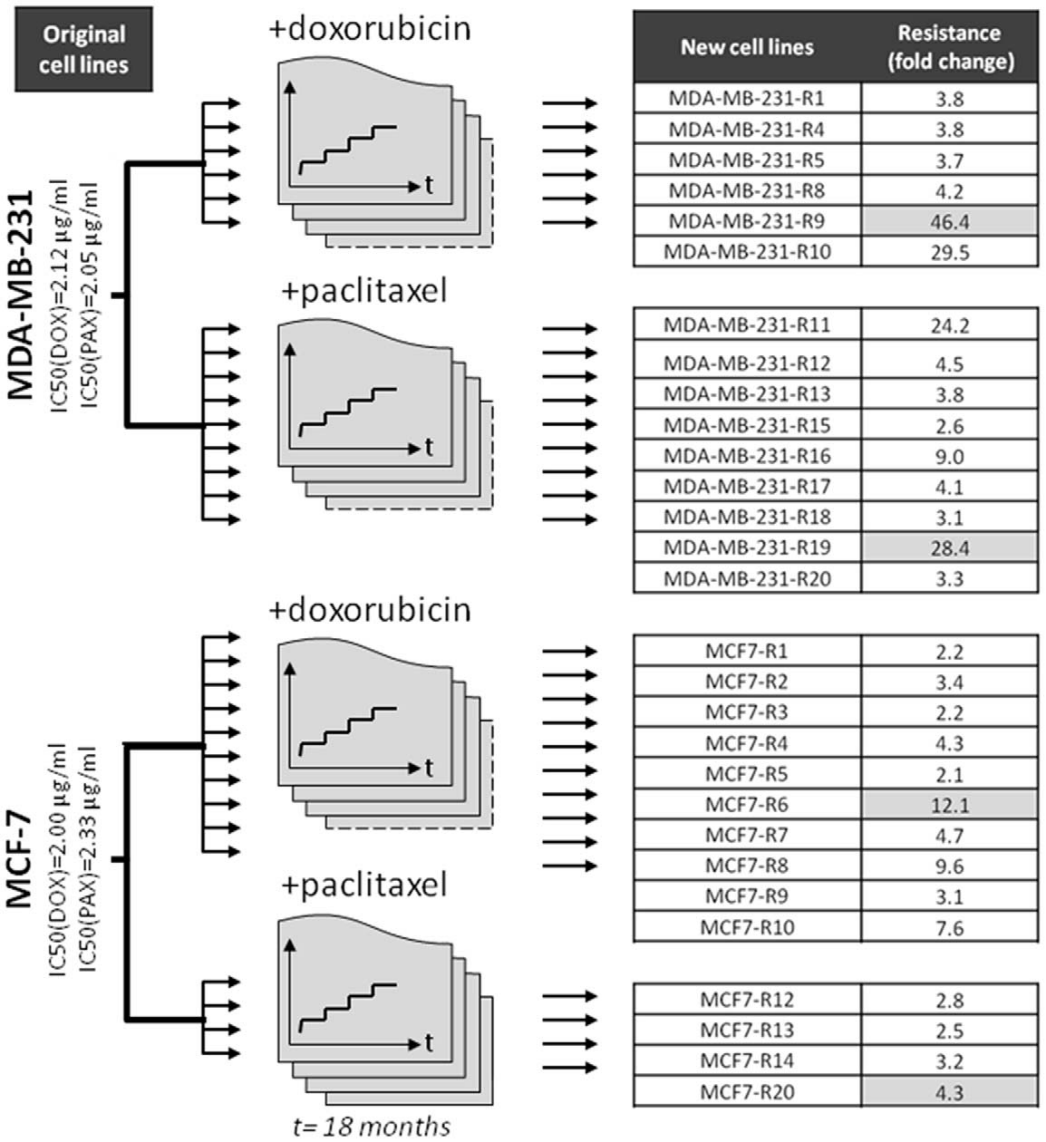
Overview of the generation of cell lines. The original cell lines were split and new cell lines were generated parallel by treatment with gradually increasing concentration of doxorubicin or paclitaxel (detailed description of the treatment protocol is in the Methods section).

At the end we obtained 29 resistant cell lines as a result of 18 months of treatment. There are 10 doxorubicin and 4 paclitaxel resistant MCF-7 cell lines, 6 doxorubicin and 9 paclitaxel resistant MDA-MB-231 cell lines. The relative resistance values compared to the original cell lines show up to 46 (average: 18.5) fold resistance against doxoxubicin and up to 28 (average: 15.4) fold resistance against paclitaxel. No cell line exhibited less than two-fold increase in the IC50 values. **Figure 1**. illustrates an overview of the process.

### Cross-resistance to four anticancer agents

**Figure 2:**
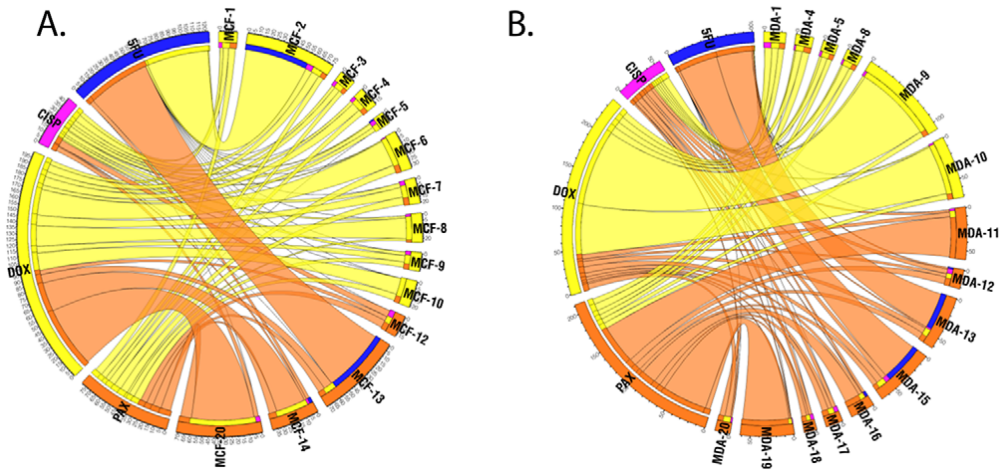
Circos plots showing the relative cross-resistance for MCF-7 derivatives (A,) and MDA-MB-231 derivatives (B,) against four chemotherapy agents. The ribbon thickness corresponds to the relative resistance. Yellow cell lines were treated with doxorubicin and orange cell lines with paclitaxel. Note the high cross-resistance against 5-fluorouracil in three paclitaxel-treated and one doxorubicin-treated cell line. In contrast, minimal cross-resistance against cisplatin can be observed. 5FU: 5-fluorouracil, CISP: cisplatin, DOX: doxorubicin, PAX: paclitaxel.

We determined the level of cross-resistance by determining the IC50 values against four widely used agents in the treatment of breast cancer (doxorubicin, paclitaxel, cisplatin and 5-fluorouracil). Although some of the cell lines exhibited significantly increased resistance against other agents as well, no significant correlation between the relative resistance levels was observed. The smallest relative resistance developed against cisplatin. Four cell lines (three of them treated with paclitaxel and one treated with doxorubicin) developed dramatic resistance against 5-fluorouracil. **Table 1**. and **Figure 2**. shows the cross-resistance properties of all cell lines against all used chemotherapy agents.

**Table 1:** Cross-resistance against doxorubicin (DOX), paclitaxel (PAX), cisplatin (CISP) and 5-fluorouracil (5-FU) in the generated breast cancer cell lines.

Cell line	Treatment	IC50 (PAX)	IC50 (DOX)	IC50 (CISP)	IC50 (5-FU)
MCF-7	Vehicle	2.33	2	2.55	0.07
MDA-MB-231	Vehicle	2.05	2.12	2.46	0.09
MDA-MB-231-R1	Doxorubicin	7.06	8.06	9.13	0.03
MDA-MB-231-R4	Doxorubicin	8.34	8.11	2.75	0.05
MDA-MB-231-R5	Doxorubicin	6.06	7.79	2.98	0.09
MDA-MB-231-R8	Doxorubicin	5.83	9	2.39	0.3
MDA-MB-231-R9	Doxorubicin	6.93	98.45	3.3	0.09
MDA-MB-231-R10	Doxorubicin	6.55	62.44	2.46	0.06
MDA-MB-231-R11	Paclitaxel	49.66	5.9	3.46	0.07
MDA-MB-231-R12	Paclitaxel	9.32	3.46	6.82	2.15
MDA-MB-231-R13	Paclitaxel	7.89	6.4	2.95	45.04
MDA-MB-231-R15	Paclitaxel	5.34	8.38	5.51	49.9
MDA-MB-231-R16	Paclitaxel	18.54	4.82	0.84	3.2
MDA-MB-231-R17	Paclitaxel	8.49	4.1	5.96	0.01
MDA-MB-231-R18	Paclitaxel	6.35	2.94	4.48	0.09
MDA-MB-231-R19	Paclitaxel	58.18	1.98	1.07	0.07
MDA-MB-231-R20	Paclitaxel	6.69	7.75	2.1	0.1
MCF-7-R1	Doxorubicin	6.88	4.46	3.14	0.4
MCF-7-R2	Doxorubicin	5.22	6.79	6.63	57.27
MCF-7-R3	Doxorubicin	4.24	4.3	3.81	0.07
MCF-7-R4	Doxorubicin	5.16	8.52	3.02	0.06
MCF-7-R5	Doxorubicin	0.7	4.19	3.45	1.61
MCF-7-R6	Doxorubicin	6.61	24.13	1.3	0.04
MCF-7-R7	Doxorubicin	6.65	9.42	4.34	0.09
MCF-7-R8	Doxorubicin	2.41	19.26	2.11	0.05
MCF-7-R9	Doxorubicin	5.34	6.28	3.1	0.05
MCF-7-R10	Doxorubicin	6.31	15.21	0.15	0.04
MCF-7-R12	Paclitaxel	6.61	4.74	6.02	0.04
MCF-7-R13	Paclitaxel	5.86	7.91	0.44	58.12
MCF-7-R14	Paclitaxel	7.48	26.94	1.16	3.95
MCF-7-R20	Paclitaxel	9.93	57.39	4.35	0.23

Although some cell lines exhibited highly marked resistance against other agents, no significant correlation between the relative resistance levels was observed. IC50: µg/ml.

### RT-PCR of pre-selected genes

**Figure 3:**
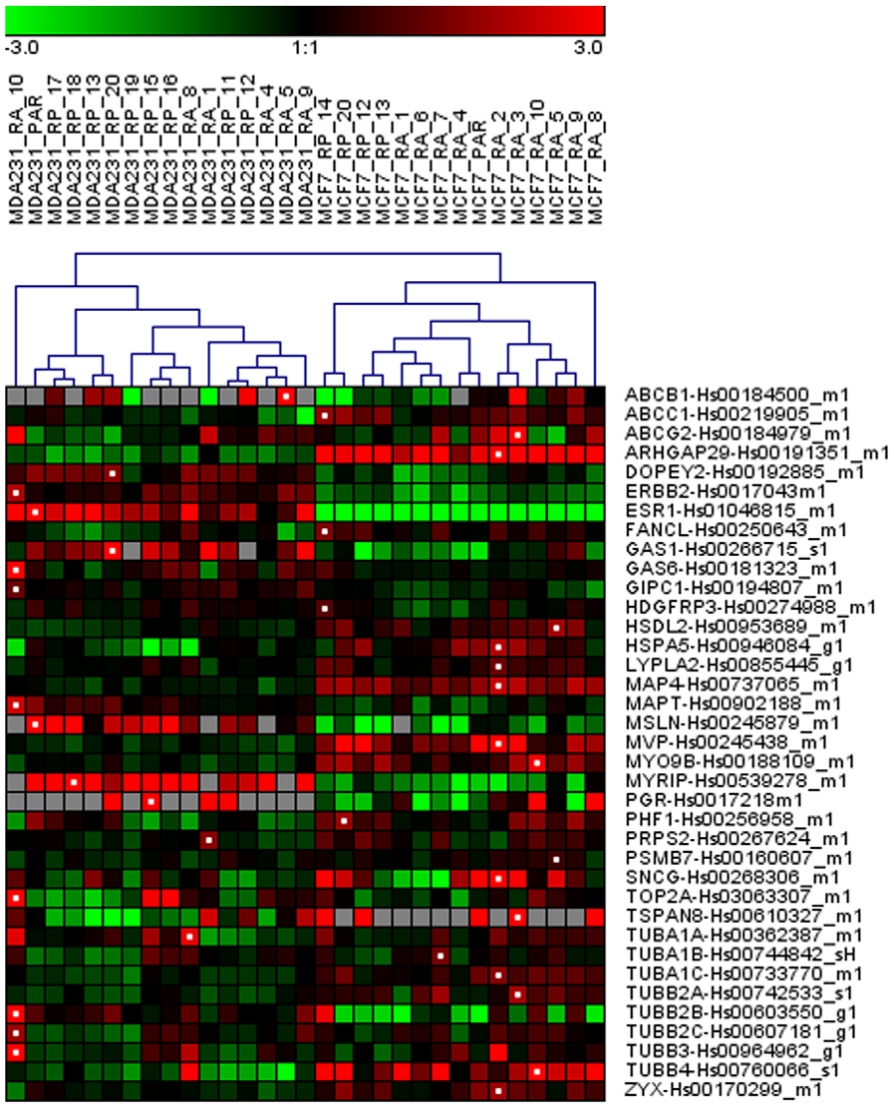
Hierarchical clustering image of the samples using the RT-PCR measured genes in both doxorubicin- and paclitaxel-resistant cell lines. Red and green: up- and down- regulated as compared to the mean of all experiments. The white dots represent the sample with the highest absolute expression. Overall only a few mechanisms are activated in one cell line to achieve resistance.

Gene expression of a set of selected genes was measured by TaqMan real-time PCR. The gene expression levels were correlated to the IC50 values using Spearman's rank correlation test. In the doxorubicin resistant cell lines the expression of TOP2A (p = 0.003) and two tubulin isoforms (TUBB2C, p = 0.003; TUBB3, p = 0.006) was correlated to the level of resistance significantly. In the paclitaxel resistant cell lines, the expression of MVP (p = 0.009), of four tubulin isoforms (TUBA1C, p = 0.003; TUBB2A, p = 0.009, TUBB4, p = 0.005) and of MAP4 (p = 0.001) correlated to resistance. ABCB1 reached a p value of 0.03 and 0.07 in the doxorubicin- and pacalitaxel-treated cell lines, respectively but these were not significant after multiple testing corrections. Hierarchical cluster visualizing the results is presented on **Figure 3**. This figure also shows the cell line with the highest absolute expression for the selected gene. Generally only a few mechanisms are activated in one cell line to achieve resistance.

### Rhodamine 123 efflux demonstrates different Pgp function in the resistant cell lines

**Figure 4:**
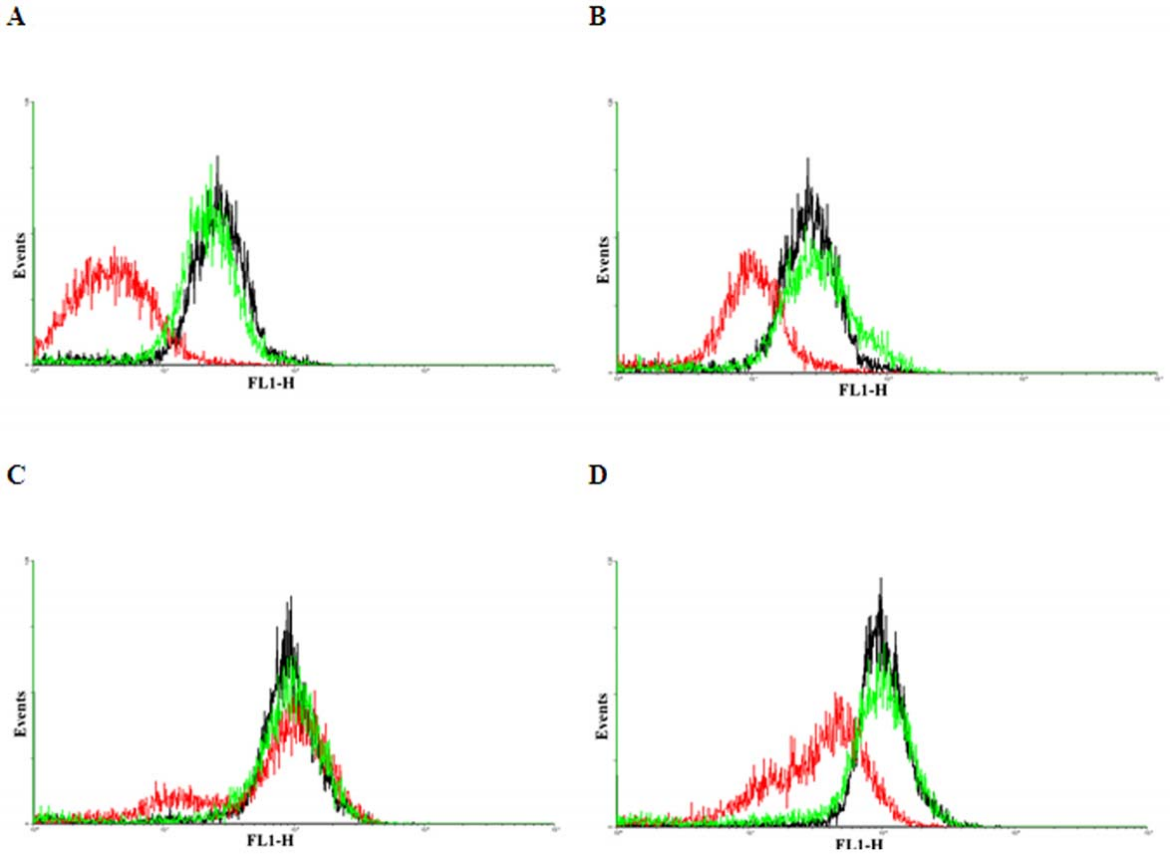
Flow cytometric analysis of rhodamin 123 stained resistant and parental MDA-MB-231 and MCF7 cell lines. (black: parental cell line, red: resistant cell line showing altered Pgp function, green: resistant cell line with normal Pgp function). **A,** MDA-MB-231-R19 (red), MDA-MB-231-R11 (green); **B,** MDA-MB-231-R1 (red), MDA-MB-231-R8 (green); **C,** MCF7-R7 (red), MCF7-R2 (green); **D,** MCF7-R20 (red), MCF7-R12 (green). Differences between the Pgp function of the resistant cell lines suggest, that alteration of the Pgp function can only explain the resistance in a few cell lines.

The ability to export drugs via Pgp-activity was assessed by FACS using the Pgp substrate rhodamine 123. The originally paclitaxel-resistant MCF-7-R20 cell lines showed the highest cross-resistance against doxorubicin. The paclitaxel-resistant MDA-MB-231-R19 showed the highest resistance against paclitaxel and also showed rhodamine efflux. Two paclitaxel-resistant cell lines (MCF-6-R5 and MCF-7-R7) showed also increased resistance against doxorubicin and cisplatin. However, a general correlation between rhodamine efflux and multidrug resistance for other agents and other cell lines could not be observed. The results of the FACS analysis for selected sets of cell lines are shown in **Figure 4**.

### Cytogenetics

**Figure 5:**
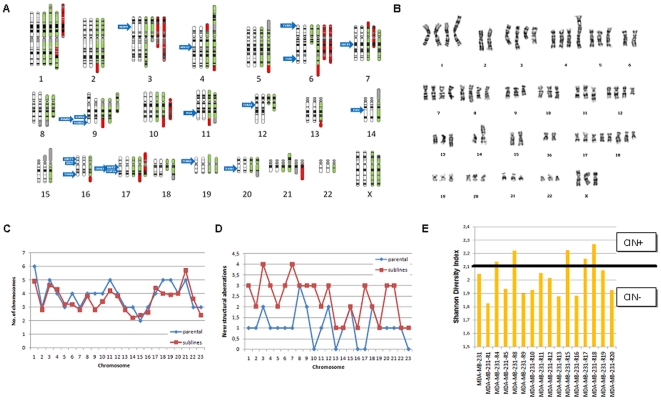
Complete overview of cytogenetic aberrations of the resistant derivatives of the MDA-MB-231 cell line. White background: normal chromosomes, green background: chromosomes of the parental MDA-MB-231 cell lines, red background: chromosomal changes in the resistant cell lines (**A,**). Position of the most important genes are marked by blue arrows. Representative karyotype of the MDA-MB-231-R18 cell line (**B,**); ploidity (**C,**) and number of new structural aberrations (**D,**) on each chromosome across the parental (blue) and all resistant (red) cell lines. Chromosomal instability (CIN) was measured computing the Shannon Diversity Index for chromosomes 3, 17 and 21 in ten different metaphases. Threshold for chromosomal instability (CIN+) was set above 2.1 (**E,**).

Cytogenetics was performed on all cell lines derived from the MDA-MB-231 cell line. The comparison of vehicle-treated and parental cell lines did not show new structural abnormalities, but the number of a few chromosomes (5, 13 and 17) changed in the vehicle treated cells compared to the parental cells. Meanwhile, there was a high number of genetic changes in the generated cell lines. The new cell lines had 60–110 chromosomes, chromosomes 1, 17 and 21 had the most copies. Based on G-band staining, the highest variability among the chromosomes were observed on chromosomes 3, 7, 17, 20 and 21. Most stable chromosomes were X, 10, 13 and 16. The most common gains were on chromosomes 15, 18 and 21. The most common deletions were 9p21 and 18q21. Two of the cell lines (MDA-MB-231-R5 and MDA-MB-231-R11) differed from the other cell lines by having a nearly tetraploid modal chromosome number. The chromosomal changes and a representative karyotypes are depicted in **Figure 5**. The main difference between the parental and the developed cell lines is in the lower number of chromosomes with higher number of new type structural rearrangements in the derivative cell lines (see **Figure 5./D**.).

### Chromosomal instability

Statistical measures of diversity typically integrate both number and abundance of chromosomes. The Shannon diversity index is preferable to other diversity measures like the Simpson's index as it is not dominated by the most frequent chromosome [32]. The average Shannon index across all cell lines was 2.03, the parental MDA-MB-231 cell line had an index of 2.05. Chromosomal instability was found in five cell lines (see **Figure 5./E**.). The CIN+ cell lines had a higher average cross-resistance as compared to the CIN- cell lines (for paclitaxel 7.1 vs. 6.7 µg/ml, cisplatin 4.2 vs. 3.5 µg/ml and 5-fluorouracil 10.1 vs. 5.1 µg/ml).

## Discussion

Starting from two breast cancer cell lines, one estrogen receptor positive and one estrogen receptor negative, using increasing concentrations of paclitaxel and doxorubicin we parallel developed 29 cell lines to establish a model similar to the evolution of the acquired drug resistance. We established a significantly higher number of resistant cell lines compared to previous studies [28] in the hope of achieving increased reliability and robustness. Interestingly, at the end the cell lines proved to be highly heterogeneous in the development of (multi) drug resistance and in confirmed genetic alterations.

In our study we focused on two questions. First, whether the multiple cell line-panel can re-identify previously clinically validated predictors of chemotherapy resistance. Our results suggest an affirmative answer and therefore validate the multiple cell line panel as a promising tool for the identification of clinically relevant biomarkers.

Second, we were interested to see how often we see multiple drug resistance emerging when selected only with a single agent. Of the 29 resistant cell lines, 25 displayed at least two-fold increased resistance against another agent, 12 of the cell lines developed over two-fold resistance against at least three agents simultaneously, and two cell lines developed at least two-fold resistance against all four agents. Therefore, while most cell lines developed resistance to at least one more drug in addition to the agent used for selection pressure, the emergence of a truly multidrug resistance phenotype seems to be a relatively rare event. This is in line with the clinical observations that tumors that developed resistance to first line therapy are still often sensitive to second or third line agents [33].

In previous studies [1], [5] we confirmed the role of members of the ATP-binding cassette family to be associated with MDR. Our FACS analysis results confirmed the correlation between ABCB1 expression and ABCB1 function, further supporting the role of ABCB1 in drug resistance. However, when compared across all the generated cell lines, members of the ABC transporter family were not always correlated to the development of resistance.

It is also notable that two genes selected for their previously described association with taxane resistance (TUBB2C ad TUBB3) were associated with doxorubicin resistance in our cell line panel, but with the opposite trend, and MVP, selected for its reported association with doxorubicin resistance, showed a significant correlation with taxane resistance.

Our study shows that the heterogeneity results in the evolution of multiple drug resistant tumor cell populations with different geno- and phenotype. The evolution of multiple resistant cell populations may require developing new treatment strategies to prevent or overcome resistance. Multiple samples from the tumor may give better insight into the clonal composition of the tumor. However it is impractical and not cost-efficient in clinical practice to perform multiple predictive tests from the same tumor, but examination of the extent of heterogeneity from a single sample may help to guide treatment decisions [34]. It is also possible, that even multiple tests would not detect a small tumor cell population playing an important role in the further development of the drug resistant tumor. A further possibility to elucidate the clonal composition of the tumor is to examine the geno- and phenotype of circulating tumor cells [35]. The better characterization of tumor heterogeneity will be vital to increase the efficacy of personalized treatment.

Finally, we must note a limitation of our cell line models, i.e. they do not represent the tumor completely as they do not take into consideration the interactions between the tumor epithelial cells and the tumor microenvironment. Meanwhile, the tumor microenvironment has been shown to be an important player in response/resistance to chemotherapy [36]. Developing resistant derivatives for example in nude mice could improve the model efficiency.

In summary, we aimed to model in vitro the emergence of drug resistance in cancer and were able to distinguish several previously clinically validated predictors. In contrast to our expectations chemoresistance evolution can emerge by only a limited number of independent mechanisms.

## Supporting Information

Table S1 (DOCX)
