# Supplementary material for: Parallel Evolution under Chemotherapy Pressure in 29 Breast Cancer Cell Lines Results in Dissimilar Mechanisms of Resistance
Source: PLoS One. 2012 Feb 2;7(2):e30804. doi: 10.1371/journal.pone.0030804 (PMC3271089; doi:10.1371/journal.pone.0030804)
Supplement: Table S1 — (DOCX) [file pone.0030804.s001.docx]

| **Paclitaxel associated genes** | **TaqMan ID** |
| --- | --- |
| tubulin alpha 1a | Hs00362387_m1 |
| tubulin, alpha 1b | Hs00744842_sH |
| tubulin, alpha 1c | Hs00733770_m1 |
| tubulin, beta 1 | Hs00258236_m1 |
| tubulin, beta 2A | Hs00742533_s1 |
| tubulin, beta 2B | Hs00603550_g1 |
| tubulin, beta 2C | Hs00607181_g1 |
| tubulin, beta 3 | Hs00964962_g1 |
| tubulin, beta 4 | Hs00893144_g1 |
| stathmin 1/oncoprotein 18 | Hs01027515_gH |
| microtubule-associated protein 4 | Hs00737065_m1 |
| microtubule-associated protein tau | Hs00902188_m1 |
| budding uninhibited by benzimidazoles 1 homolog beta (yeast) | Hs01084828_m1 |
| MAD2 mitotic arrest deficient-like 1 (yeast) | Hs03063324_g1 |
| polo-like kinase 2 (Drosophila) | Hs01573415_g1 |
| ATP-binding cassette, sub-family C (CFTR/MRP), member 2 | Hs00166123_m1 |
| ATP-binding cassette, sub-family C (CFTR/MRP), member 1 | Hs00219905_m1 |
| ATP-binding cassette, sub-family C (CFTR/MRP), member 10 | Hs00375701_m1 |
| **Doxorubicin associated genes** | **TaqMan ID** |
| ATP-binding cassette, sub-family C (CFTR/MRP), member 1 | Hs00219905_m1 |
| topoisomerase (DNA) II alpha 170kDa | Hs03063307_m1 |
| ATP-binding cassette, sub-family B (MDR/TAP), member 1 | Hs00184500_m1 |
| major vault protein | Hs00245438_m1 |
| ATP-binding cassette, sub-family G (WHITE), member 2 | Hs00184979_m1 |
| superoxide dismutase 2, mitochondrial | Hs01553554_m1 |
| glutathione S-transferase pi 1 | Hs00943351_g1 |
| Fas (TNF receptor superfamily, member 6) | Hs00163653_m1 |
| heat shock 70kDa protein 5 (glucose-regulated protein, 78kDa) | Hs00946084_g1 |
| X-box binding protein 1 | Hs02856596_m1 |
| hypoxia inducible factor 1, alpha subunit | Hs00153153_m1 |
